# Proteomic analysis reveals dual requirement for Grb2 and PLCγ1 interactions for BCR-FGFR1-Driven 8p11 cell proliferation

**DOI:** 10.18632/oncotarget.28228

**Published:** 2022-05-11

**Authors:** Malalage N. Peiris, April N. Meyer, Dalida Warda, Alexandre Rosa Campos, Daniel J. Donoghue

**Affiliations:** ^1^Department of Chemistry and Biochemistry, University of California San Diego, La Jolla, CA 92037, USA; ^2^Proteomics Facility, Sanford Burnham Prebys Medical Discovery Institute, La Jolla, CA 92037, USA; ^3^UCSD Moores Cancer Center, University of California San Diego, La Jolla, CA 92037, USA

**Keywords:** oncogenic fusion protein, chromosomal translocation, protein interactome, phosphoproteome, stem cell leukemia/lymphoma

## Abstract

Translocation of Fibroblast Growth Factor Receptors (FGFRs) often leads to aberrant cell proliferation and cancer. The BCR-FGFR1 fusion protein, created by chromosomal translocation t(8;22)(p11;q11), contains Breakpoint Cluster Region (BCR) joined to Fibroblast Growth Factor Receptor 1 (FGFR1). BCR-FGFR1 represents a significant driver of 8p11 myeloproliferative syndrome, or stem cell leukemia/lymphoma, which progresses to acute myeloid leukemia or T-cell lymphoblastic leukemia/lymphoma. Mutations were introduced at Y177F, the binding site for adapter protein Grb2 within BCR; and at Y766F, the binding site for the membrane associated enzyme PLCγ1 within FGFR1. We examined anchorage-independent cell growth, overall cell proliferation using hematopoietic cells, and activation of downstream signaling pathways. BCR-FGFR1-induced changes in protein phosphorylation, binding partners, and signaling pathways were dissected using quantitative proteomics to interrogate the protein interactome, the phosphoproteome, and the interactome of BCR-FGFR1. The effects on BCR-FGFR1-stimulated cell proliferation were examined using the PLCγ1 inhibitor U73122, and the irreversible FGFR inhibitor futibatinib (TAS-120), both of which demonstrated efficacy. An absolute requirement is demonstrated for the dual binding partners Grb2 and PLCγ1 in BCR-FGFR1-driven cell proliferation, and new proteins such as ECSIT, USP15, GPR89, GAB1, and PTPN11 are identified as key effectors for hematopoietic transformation by BCR-FGFR1.

## INTRODUCTION

Over the last half century, chromosomal translocations encoding functional oncogenic proteins have been identified as drivers of multiple cancers, and account for 20% of all malignant neoplasms [1, 2]. With the emergence of personalized medicine and cancer genome sequencing, the characterization of these oncogenic fusions created by chromosomal translocations – which serve as drivers of specific cancers – is vital to advance therapeutic methods and improve outcomes.

Genomic studies have revealed the presence of many specific RTK fusion proteins as drivers of blood cancers [3]. In particular, fibroblast growth factor receptors (FGFRs), a subfamily of RTKs, have been identified as recurrent translocation partners in both solid and hematologic malignancies [4]. Constitutively activated FGFR1 fusion proteins give rise to 8p11 myeloproliferative syndrome (EMS), also known as stem cell leukemia/lymphoma (SCLL), which can progress to acute myeloid leukemia (AML) or T-cell acute lymphoblastic leukemia lymphoma (T-ALL), dependent on the fusion partner gene [5, 6]. Patients positive for FGFR1-driven SCLL often present with eosinophilia and have a poor prognosis as these fusions are not respondent to first generation tyrosine kinase inhibitor (TKI) therapies, and the one-year overall survival from time of diagnosis is 43% for SCLL patients [5, 7]. Although both ponatinib and pemigatinib have been used to treat SCLL with mixed results, hematopoietic stem cell transplantation remains the only known curative option for SCLL patients and few alternative treatment plans exist for those who are either awaiting or are unable to receive transplantation [8]. The poor prognosis and lack of molecular targeted therapies highlights SCLL as a critically unmet medical need.

This work focuses on the t(8;22)(p11;q11) chromosomal translocation which creates the Breakpoint Cluster Region-Fibroblast Growth Factor Receptor 1 (BCR-FGFR1) fusion protein. This fusion protein retains the coiled-coil dimerization/oligomerization domain and partial RhoGEF domain contributed by BCR, and a tyrosine kinase domain contributed by FGFR1. Our recent work demonstrated the importance of the Hsp90 protein chaperone complex for BCR-FGFR1 driven oncogenic activation, together with the importance of several salt bridges for stabilization of the coiled-coil dimerization domain of BCR [9].

Earlier work examining two FGFR1-containing fusion proteins, BCR-FGFR1 and ZNF198-FGFR1, provided important insights into mechanisms of cancer progression; specifically, this work identified the importance of the phospholipase PLCγ1 binding site at Y766 in the ZNF198-FGFR1 fusion, and the importance of the small adapter protein Grb2 binding site at Y177 in BCR-FGFR1 for progression of myeloproliferative disease in murine models [10]. From this work, they concluded that PLCγ1 represents a critical downstream pathway for ZNF198-FGFR1-induced disease, and that Grb2 activation was important for BCR-FGFR1 in the induction of CML-like leukemia in mice [10].

Building from these advances, our current work examines mutations in the PLCγ1 and Grb2 binding sites individually and, importantly, when combined together in a double mutant within BCR-FGFR1. Importantly, our work finds that this Grb2 and PLCγ1 binding site double mutant is no longer biologically active. We exploit quantitative proteomic analyses to identify crucial protein-protein interactions necessary for BCR-FGFR1 activation. Thus, we are able to demonstrate a dual requirement for Grb2 and PLCγ1 for BCR-FGFR1-mediated oncogenic cell proliferation. We extensively profiled the differences in cell signaling between BCR-FGFR1 and the non-biologically active mutants BCR(Y177F)-FGFR1(Y766F), and BCR(Y177F)-FGFR1(K656E/Y766F), containing both Grb2 and PLCγ1 interaction site mutations, through proteomics analysis to elucidate the BCR-FGFR1 total proteome, the phosphoproteome, and protein interactome. This systemic study reveals the multisubstrate docking protein, Gab1, and the protein tyrosine phosphatase, PTPN11 (Shp2), as likely downstream targets of Grb2 and PLCγ1 in BCR-FGFR1-driven SCLL. Furthermore, we identified PLCγ1 as potential therapeutic target to treat BCR-FGFR1 mediated SCLL using the PLCγ1 inhibitor U73122, and show that futibatinib, an irreversible FGFR inhibitor, suppresses downstream signaling and cell transformation. These data unravel essential roles of Grb2 and PLCγ1 in BCR-FGFR1 mediated oncogenic growth and suggest the importance of further investigation into PLCγ1 as a potential therapeutic target in treating SCLL.

## RESULTS

### BCR-FGFR1 requires Grb2 and PLCγ1 interaction for cell transformation and proliferation

During RTK-mediated signal transduction, Grb2, a small adapter protein, associates with SOS (son of sevenless), leading to Ras activation. Furthermore, the enzyme PLCγ1, a protein involved in cell growth and proliferation, has been known to play a role in cancer progression, yet the role of PLCγ1 in BCR-FGFR1-mediated malignancies is undetermined [11].

**Figure 1:**
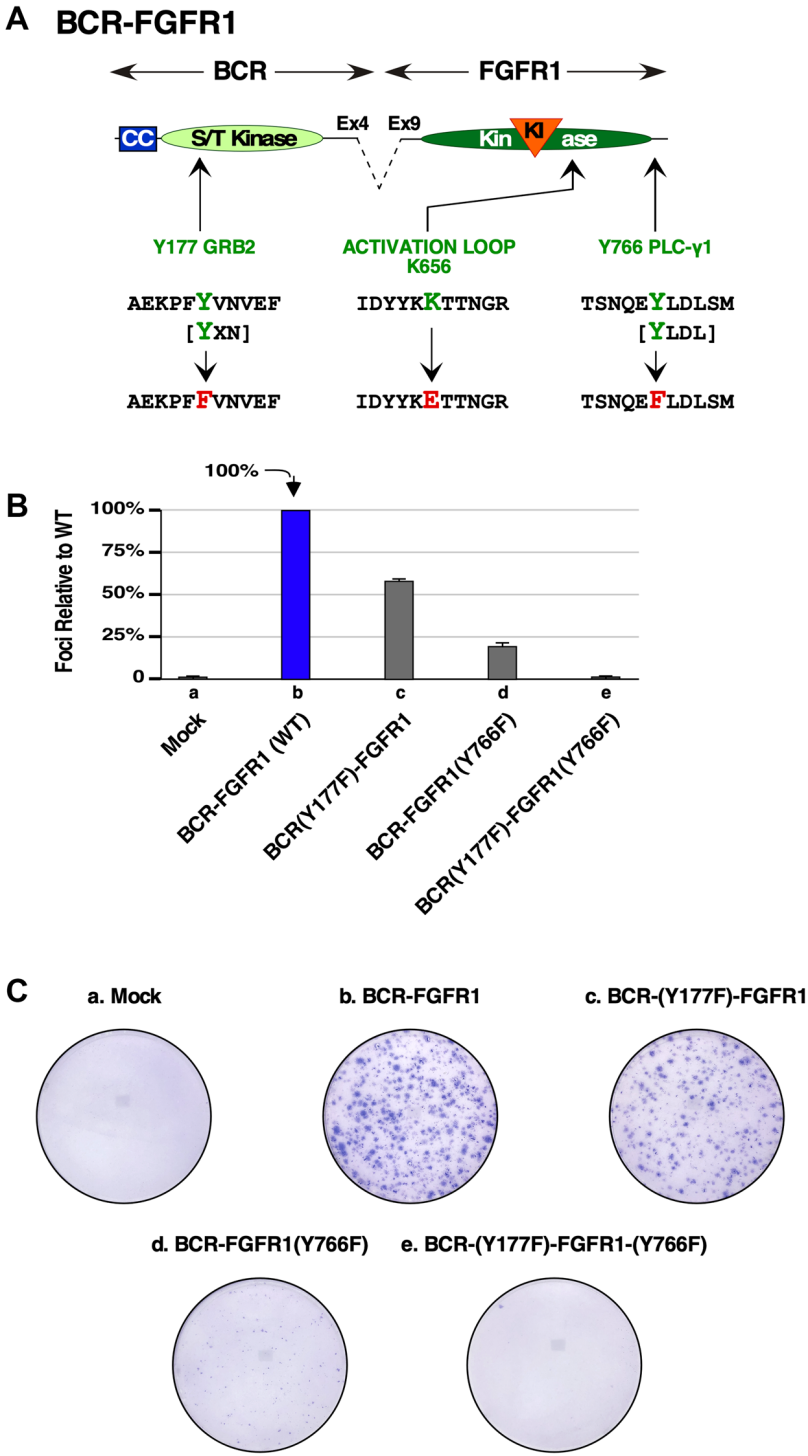
Biological assays of BCR-FGFR1 or the BCR-FGFR1 derivatives lacking Grb2 or PLCγ1 interaction sites. (**A**) A schematic of amino acid mutations made to ablate the Grb2 and PLCγ1 interaction sites in BCR-FGFR1. The kinase activating mutation K656E is also shown. (**B**) A graph of NIH3T3 focus formation relative to BCR-FGFR1. Each experiment was performed a minimum of 3 times, and standard error of the mean (SEM) is shown. (**C**) Pictures of representative focus assay plates stained with Giemsa to visualize foci. Mock cells are used as a negative control.

We constructed BCR-FGFR1 derivatives containing single mutations to abolish the Grb2 and PLCγ1 interaction sites, and BCR(Y177F)-FGFR1(Y766F), containing a double mutation abolishing both interaction sites (Figure 1A). These were assayed for NIH3T3 focus formation (Figure 1B and 1C). NIH3T3 cells expressing BCR(Y177F)-FGFR1 exhibited nearly a 50% decrease in focus forming ability, while cells expressing BCR-FGFR1(Y766F) showed an 80% (Figure 1B and 1C). Interestingly, the double mutant BCR(Y177F)-FGFR1(Y766F) completely abolished focus formation in this assay. Although the use of NIH3T3 cells, a murine fibroblast cell line, may be criticized as a proxy for hematopoietic cell cancer, nevertheless, this assay has routinely served as a useful biological readout for the assay of many different oncogenic fusion proteins [9, 12, 13].

### STAT3 signaling along with Grb2 and PLCγ1 association are necessary for BCR-FGFR1 mediated cell growth

**Figure 2:**
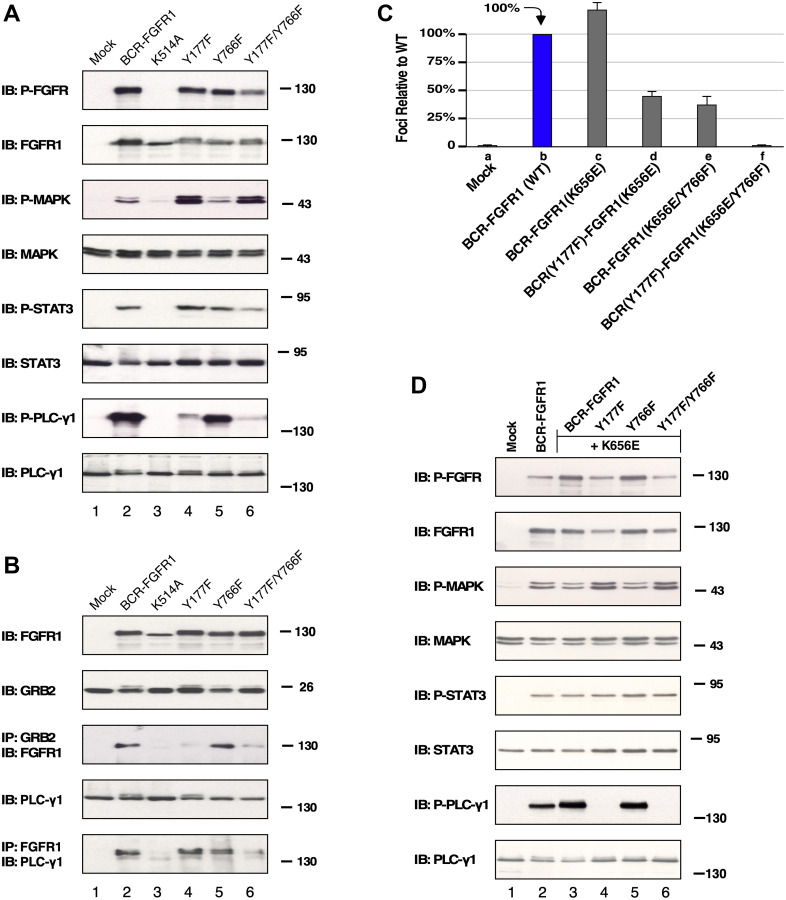
HEK293T cell lysate expressing BCR-FGFR1 or its derivatives subject to immunoblot analysis. (**A**) Downstream pathways potentially activated by either BCR-FGFR1, a kinase inactivated BCR-FGFR1(K514A), BCR(Y177F)-FGFR1, BCR-FGFR1(Y766F), or BCR(Y177F)-FGFR1(Y766F) were examined. All pathways were detected by anti-sera directed towards each phosphorylated protein as shown. Blotting for total protein shown below each activated panel. (**B**) Protein interactions are shown by immunoprecipitation with antisera for Grb2 or FGFR1 followed by immunoblotting with anti-sera against either FGFR1 or PLCγ1 to detect protein interactions for BCR-FGFR1. Each experiment was performed a minimum of 3 times. (**C**) Graph of focus formation by BCR-FGFR1(K656E) and its derivatives in NIH3T3 cells. Each experiment was performed a minimum of 3 times, and standard error of the mean (SEM) is shown. (**D**) HEK293T cell lysate expressing BCR-FGFR1(K656E) or its derivatives subjected to immunoblot analysis. All pathways were detected by anti-sera directed towards each phosphorylated protein as shown, followed directly below by blotting for each total protein. Each experiment was performed a minimum of 3 times, and standard error of the mean (SEM) is shown.

The cell signaling differences between BCR-FGFR1 and the non-transforming derivative, BCR(Y177F)-FGFR1(Y766F), remain unclear, particularly since this mutant retains tyrosine kinase activity contributed by FGFR1 [9]. Signaling analyses were performed in HEK293T cells, as they have previously been used in FGFR signal transduction and protein phosphorylation studies [12]. HEK293T cells expressing either BCR-FGFR1, a kinase-dead variant BCR-FGFR1(K514A), single mutants, or the non-transforming double mutant BCR(Y177F)-FGFR1(Y766F), were analyzed for cell signaling differences by immunoblotting. HEK293T cells expressing BCR-FGFR1 display activation of MAPK, STAT3, and PLCγ1 pathways, while BCR-FGFR1(K514A), containing a kinase-inactivating mutation, was unable to activate downstream pathways (Figure 2A). HEK293T cells expressing the non-transforming BCR(Y177F)-FGFR1(Y766F) displayed a substantial decrease in STAT3 signaling and nearly total ablation of PLCγ1 phosphorylation, even while retaining FGFR1 activation loop phosphorylation (Tyr653/Tyr654) (Figure 2A, lane 6). While BCR(Y177F)-FGFR1 and BCR-FGFR1(Y766F) single mutants retained similar FGFR1 activation loop phosphorylation levels and STAT3 activation as the BCR-FGFR1 fusion, each of these varied in MAPK and PLCγ1 activation.

Additionally, cells expressing BCR(Y177F)-FGFR1(Y766F) were unable to interact with Grb2 and PLCγ1, as seen through immunoprecipitation analyses followed by immunoblot analysis (Figure 2B). BCR-FGFR1(K514A), the kinase-inactive mutant, was unable to associate with either Grb2 or PLCγ1, suggesting that receptor kinase activity leading to tyrosine phosphorylation is required for this protein-protein interaction (Figure 2B, lane 3). These data suggest that BCR-FGFR1 may rely on the Jak/STAT pathway and interactions with Grb2 and PLCγ1 for cell proliferation, as BCR(Y177F)-FGFR1(Y766F) displays low levels of STAT3 activation, and minimal association with Grb2 and PLCγ1 (Figure 2A and 2B). Furthermore, MAPK activation may be inconsequential for BCR-FGFR1-driven oncogenesis, as cells expressing BCR(Y177F)-FGFR1(Y766F) exhibited increased levels of MAPK phosphorylation despite the inability of this variant to transform NIH3T3 cells (Figure 1B).

### Kinase-activating mutations in BCR-FGFR1 do not overcome a dual Grb2 and PLCγ1 interaction requirement

Kinase-activating mutations and gatekeeper mutations are commonly found in patients receiving TKI treatment [14]. Therefore, we introduced a kinase-activating K656E mutation (Figure 1A) to determine if a constitutively activated kinase would alter the potential requirement for Grb2 and PLCγ1 interactions with BCR-FGFR1 for cell transformation and signal cascade activation. The K656E mutation lies within the “YYKK” activation loop sequence in FGFR1 and is an activating mutation found in cancers as well as developmental disorders [4, 15, 16].

When assayed for focus formation, cells expressing BCR-FGFR1, or the kinase-activated variant, BCR-FGFR1(K656E), were biologically active and generated foci. However, when the double mutant was combined with the kinase-activating mutation, the resulting BCR(Y177F)-FGFR1(K656E/Y766F) was unable to transform NIH3T3 cells (Figure 2C). Cells expressing the non-transforming triple mutant, BCR(Y177F)-FGFR1(K656E/Y766F), containing a deficiency in both Grb2 and PLCγ1 interaction sites along with the kinase-activating K565E mutation, displayed a lack of PLCγ1 phosphorylation while maintaining FGFR1 activation loop phosphorylation (Figure 2D, lane 6). Additionally, these cells displayed increased levels of MAPK phosphorylation, similar to BCR(Y177F)-FGFR1(Y766F), despite the inability of either of these variants to transform NIH3T3 cells (Figure 2A and 2D). These data suggest that this kinase-activating mutation is unable to overcome the need for protein-protein interactions between BCR-FGFR1 with both Grb2 and PLCγ1 for oncogenic growth, highlighting the importance of these interactions as plausible therapeutic targets.

### Characterization of the BCR-FGFR1 protein interactome and phospho-proteome

Examining the protein interactome and phospho-proteome of various oncogenes have led to the identification of important biomarkers and therapeutic targets in cancer [17–19]. Recent studies have utilized proteomic approaches to determine differences in cell signaling between BCR-ABL p210 and p190 isoforms [20]. We employed quantitative mass spectrometry to characterize the BCR-FGFR1 mediated protein interaction network, or interactome, as well as the BCR-FGFR1 mediated phospho-proteome. For these proteomic studies, four biological replicates of each sample were included to achieve statistical significance. Of importance, the inclusion of the biologically inactive, but kinase-activated mutant, BCR(Y177F)-FGFR1(K656E/Y766F), allowed the elimination of many interacting and phosphorylated peptides that might otherwise appear as authentic hits.

### Interactome analysis

**Figure 3:**
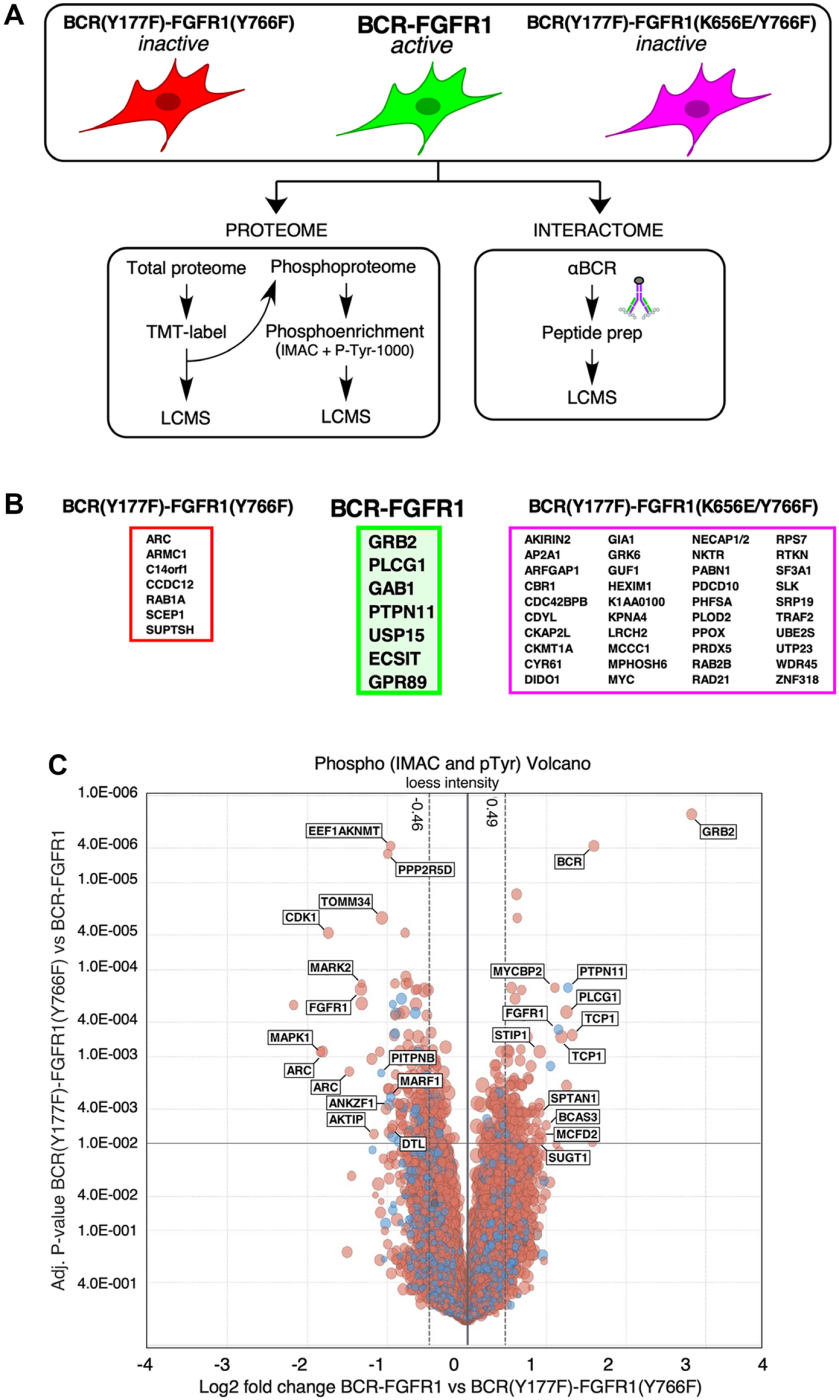
LC-MS/MS determination of the protein interactome and phospho-proteome of BCR-FGFR1 and its inactive derivatives. (**A**) A schematic of the workflow used for LC/MS. HEK293T cells expressing either BCR-FGFR1 or biologically inactive derivatives were subjected to proteome and phospho-proteome analysis and, separately, analyzed for interactome analysis. (**B**) Identified interacting proteins with either BCR-FGFR1, outlined in green, or the biologically inactive mutants, outlined in either red or magenta. (**C**) A volcano plot representation of phosphorylated proteins in BCR(Y177F)-FGFR1(Y766F) compared to BCR-FGFR1. This plot is normalized to log2 fold change and the respective adjusted *p*-values between BCR-FGFR1 and BCR(Y177F)-FGFR1(Y766F). Four independent biological replicates were used for each sample. For all datasets, results were initially normalized against the kinase-dead BCR-FGFR1(K514A), for which quadruplicate samples were analyzed in parallel with the active mutant, BCR-FGFR1, and the two biologically inactive mutants, BCR(Y177F)-FGFR1(Y766F) and BCR(Y177F)-FGFR1(K656E/Y766F). Dashed vertical lines represent +/− 1 standard deviation from the mean.

HEK293T cells expressing either BCR-FGFR1, BCR(Y177F)-FGFR1(Y766F), BCR(Y177F)-FGFR1(K656E/Y766F), or a kinase-inactive BCR-FGFR1(K514A) were lysed in Tandem Affinity Purification (TAP) buffer (Figure 3A). Immunopurification of BCR-FGFR1 complexes was achieved using an antiserum directed against the N-terminal BCR domain, and immune complexes were collected on protein A/G magnetic beads.

**Figure 4:**
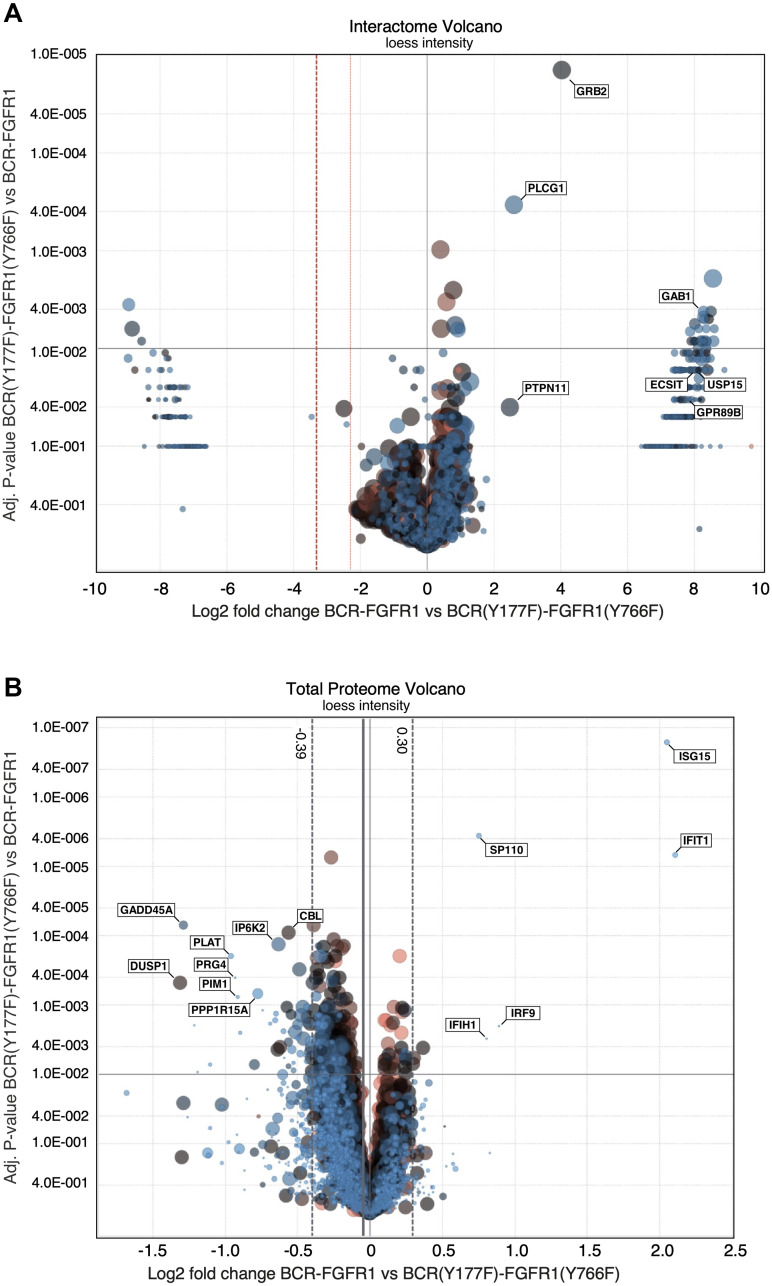
LC/MS-MS interactome and proteome volcano plots comparing BCR-FGFR1 with BCR(Y177F)-FGFR1(K656E). (**A**) A volcano plot representation of interacting proteins in BCR(Y177F)-FGFR1(Y766F) compared to BCR-FGFR1. This plot is normalized to log2 fold change and the respective adjusted *p*-values between BCR-FGFR1 and BCR(Y177F)-FGFR1(Y766F). (**B**) A volcano plot representation of the total proteome in BCR(Y177F)-FGFR1(Y766F) compared to BCR-FGFR1. This plot is normalized to log2 fold change and the respective adjusted *p*-values between BCR-FGFR1 and BCR(Y177F)-FGFR1(Y766F). Four independent biological replicates were used for each sample. For all datasets, results were initially normalized against the kinase-dead BCR-FGFR1(K514A), for which quadruplicate samples were analyzed in parallel with the active mutant, BCR-FGFR1, and the two biologically inactive mutants, BCR(Y177F)-FGFR1(Y766F) and BCR(Y177F)-FGFR1(K656E/Y766F). Dashed vertical lines represent +/− 1 standard deviation from the mean.

This interactome analysis detected over 3000 unique BCR-FGFR1 derivative complexes. To subsequently identify the interactome differences between BCR-FGFR1 and the non-biologically active mutants, interacting protein hits were screened against interactions with the kinase inactive BCR-FGFR1(K514A) mutant. Each interacting protein presented in this data was detected in at least three out of four biological replicates (Figures 3B and 4A).

BCR-FGFR1 preferentially forms protein complexes with only seven proteins, including PTPN11 (Shp2), Gab1, ECSIT, USP15, and GPR89 in addition to Grb2 and PLCγ1, when compared to the biologically inactive mutants (Figures 3B and 4A). Of these identified complexes, BCR-FGFR1 interactions with PTPN11 and Gab1 are particularly interesting. PTPN11 is a well-studied tyrosine phosphatase, known to modulate oncogenic signaling pathways downstream of Grb2, while Gab1 is an adapter protein associated with Grb2, and known to activate signal transduction pathways [21, 22]. Furthermore, ECSIT is an adapter protein known to activate the NF-κB signaling pathway, and USP15 is a deubiquitinating enzyme (DUB) responsible for ubiquitin chain cleavage on known substrates, ultimately leading to cancer cell survival [23, 24]. GPR89, or G Protein-Coupled Receptor 89A, represents an effector for the RAS family member RABL3 in hematopoietic cells [25]. These data support previous studies demonstrating that PTPN11 inhibition reduces BCR-FGFR1-driven cell viability and leads to suppression of leukemogenesis in mice [26]. Discovery of the novel interacting proteins ECSIT and USP15 as potential targets in BCR-FGFR1 mediated cell growth will require further investigation to determine their roll in SCLL progression.

### Phospho-proteome analysis

We also wished to characterize the BCR-FGFR1 induced total proteome and phospho-proteome to further understand cell signaling differences between the fusion and the biologically inactive mutants. HEK293T cells expressing either BCR-FGFR1 or its derivatives were harvested in PBS, labeled with a tandem mass tag (TMT) [27], and subjected to IMAC and CST Y1000 phospho-enrichment prior to LC-MS/MS detection (Figure 3A). The resulting phosphopeptides were then combined to provide greater overall coverage of the BCR-FGFR1 phosphoproteome.

This phospho-proteome analysis method resulted in the detection of over 5,000 phosphorylated proteins (Figure 3C). As expected, BCR-FGFR1 demonstrated an increase in Grb2 and PLCγ1 phosphorylation, when compared to its biologically inactive mutants; furthermore, an increase in PTPN11 and TCP1 phosphorylation was also detected in BCR-FGFR1 (Figure 3C). Of note, PTPN11 (Shp2) preferentially formed protein complexes with BCR-FGFR1 as seen through the interactome data (Figures 3B and 4A). BCR-FGFR1 stimulates TCP1 phosphorylation, a protein involved in the TRiC chaperone complex [28], suggesting that TCP1 mediated protein folding may play a role in the regulation of the BCR-FGFR1 oncoprotein. The inactive BCR-FGFR1 mutant also demonstrated an increase in MAPK1, MARK2, and CDK1 phosphorylations (Figure 3C, Table 1A).

**Table 1A:** Phospho-sites for BCR-FGFR1 associated phospho-proteome

Phosphorylated sites (upregulated)	*P* Value
GRB2_Y209	5.99E-11
BCR_Y177	3.40E-10
TCP1_S544,TCP1_S551	9.29E-07
PTPN11_Y63,PTPN11_Y66	1.06E-07
TCP1_S544,TCP1_Y545,TCP1_S551	9.37E-06
PLCG1_Y428	3.76E-07
FGFR1_S762,FGFR1_Y776	7.75E-07
TCP1_S544,TCP1_Y545	8.56E-07
MYCBP2_S2873	1.05E-07
BCAS3_S886	6.90E-05
MCFD2_Y135	9.98E-05
SUGT1_Y90	1.28E-04
SPTAN1_Y1261	3.93E-05
STIP1_Y376	2.02E-06
**Phosphorylated sites (downregulated)**	***P* Value **
MAPK1_T181,MAPK1_T185	3.55E-06
ARC_Y14	2.00E-06
CDK1_S39	9.87E-09
ARC_Y78	4.49E-06
MARK2_S40,MARK2_Y53	7.55E-08
AKTIP_S16	1.08E-04
PITPNB_S267	8.51E-06
TOMM34_S186	2.02E-07
PITPNB_S267	4.86E-06
PPP2R5D_S60,PPP2R5D_S62	1.07E-07
MARF1_S536	3.34E-05
EEF1AKNMT_S267	7.65E-09
MAPK1_T190	1.29E-05
MARF1_S536	1.10E-05
FGFR1_S450,FGFR1_S451,FGFR1_S461	1.05E-07
DTL_S717	4.05E-05
ANKZF1_S47,ANKZF1_S51,ANKZF1_S56	1.59E-05

The BCR-FGFR1 associated phospho-proteome demonstrates an increase in proteins associated with catalytic activity, signal transduction, and cell communication, as seen through gene ontology analyses (Table 1B). Overall, these data demonstrate that the BCR-FGFR1 phospho-proteome may be driven by Grb2, PLCγ1, and PTPN11 mediated signaling cascades, with the ultimate result of cell proliferation.

**Table 1B:** Upregulated GO (Gene Ontology) functions for BCR-FGFR1 associated phospho-proteome

GO Term	Description	*P*-value
GO: 0050790	Regulation of catalytic activity	9.76E-06
GO: 0097485	Neuron projection guidance	4.70E-05
GO: 0007411	Axon guidance	4.70E-05
GO: 0007166	Cell surface receptor signaling pathway	6.30E-05
GO: 0035556	Intracellular signal transduction	6.93E-05
GO: 0065009	Regulation of molecular function	8.25E-05
GO: 0007165	Signal transduction	1.09E-04
GO: 0019221	Cytokine-mediated signaling pathway	1.25E-04
GO: 0051336	Regulation of hydrolase activity	2.24E-04
GO: 0002252	Immune effector process	2.60E-04
GO: 0007167	Enzyme linked receptor protein signaling pathway	2.97E-04
GO: 0007169	Transmembrane receptor protein tyrosine kinase signaling pathway	3.04E-04
GO: 0010646	Regulation of cell communication	3.17E-04
GO: 0043085	Positive regulation of catalytic activity	6.49E-04
GO: 0007173	Epidermal growth factor receptor signaling pathway	7.30E-04
GO: 0051338	Regulation of transferase activity	8.96E-04
GO: 0023051	Regulation of signaling	9.42E-04
GO: 0042058	Regulation of epidermal growth factor receptor signaling pathway	9.49E-04

### Total proteome analysis

The total proteome was analyzed to identify differences in protein expression that contribute to the activity of BCR-FGFR1. The BCR-FGFR1 proteome is associated with an increase in expression of several proteins, notably, ISG15, IFIT1, IRF9 and SP110, which are interferon response genes associated with JAK/STAT signaling (Table 1C, Figure 4B) [29, 30]. Overexpression of these proteins may explain the increase in STAT3 activation seen in BCR-FGFR1 compared to biologically inactive derivatives. Furthermore, the proteomes of both BCR(Y177F)-FGFR1(Y766F) and BCR(Y177F)-FGFR1(K656E/Y766F) are associated with an increase in expression of 44 proteins and with a decrease in 8 proteins when compared to BCR-FGFR1 (Figure 4B). Of these, GADD45A is a well characterized TP53 effector and stress-induced protein shown to induce overactivation of the MAPK pathway, resulting ultimately in apoptosis [31]. The overexpression of GADD45A may explain the increase in phosphorylated MAPK signaling in the BCR-FGFR1 biologically inactive mutants as seen by immunoblotting (Figure 2A and 2D) and phospho-proteome analysis (Figure 3C, Table 1A). Overall, the total proteome of the BCR-FGFR1 fusion demonstrates an increase in cytokine stimulus and interferon response genes, while the biologically inactive mutants demonstrate an increase in apoptotic pathways, negative regulation of kinase signaling, and positive regulation of ubiquitination, as seen through gene ontology analyses (Table 1C).

**Table 1C:** GO functions for BCR-FGFR1 associated proteome

GO Process (upregulated)	Proteins
GO_CELLULAR_RESPONSE_TO_CYTOKINE_STIMULUS	IRF9
	IFIT1
	ISG15
GO_CYTOKINE_MEDIATED_SIGNALING_PATHWAY	IRF9
	IFIT1
	ISG15
GO_RESPONSE_TO_TYPE_I_INTERFERON	IRF9
	IFIT1
	ISG15
**GO Process (downregulated)**	**Proteins**
GO_APOPTOTIC_SIGNALING_PATHWAY	CDKN1A
	CHAC1
	DDIT3
	DDIT4
	E2F2
	PPP1R15A
	TRIB3
GO_CELL_CYCLE_ARREST	CDKN1A
	DDIT3
	DUSP1
	GADD45A
	MYC
	PPP1R15A
GO_INTRINSIC_APOPTOTIC_SIGNALING_PATHWAY	CDKN1A
	CHAC1
	DDIT3
	DDIT4
	E2F2
	PPP1R15A
	TRIB3
GO_NEGATIVE_REGULATION_OF_INTRACELLULAR_SIGNAL_TRANSDUCTION	ATF3
	DDIT3
	DDIT4
	DUSP1
	MYC
GO_NEGATIVE_REGULATION_OF_KINASE_ACTIVITY	CDKN1A
	DUSP1
	GADD45A
	TRIB3
GO_NEGATIVE_REGULATION_OF_PHOSPHORYLATION	ATF3
	CDKN1A
	DDIT4
	DUSP1
	GADD45A
	MYC
	PPP1R15A
	TRIB3
GO_PROTEIN_UBIQUITINATION	CBL
	KLHL21
	KLHL28
	TTC3
	WSB1
GO_PROTEIN_UBIQUITINATION_INVOLVED_IN_UBIQUITIN_DEPENDENT_PROTEIN_CATABOLIC_PROCESS	CBL
	KLHL21
	KLHL28

### Examination of PLCγ1 and Grb2 mutations on hematopoietic cell proliferation

**Figure 5:**
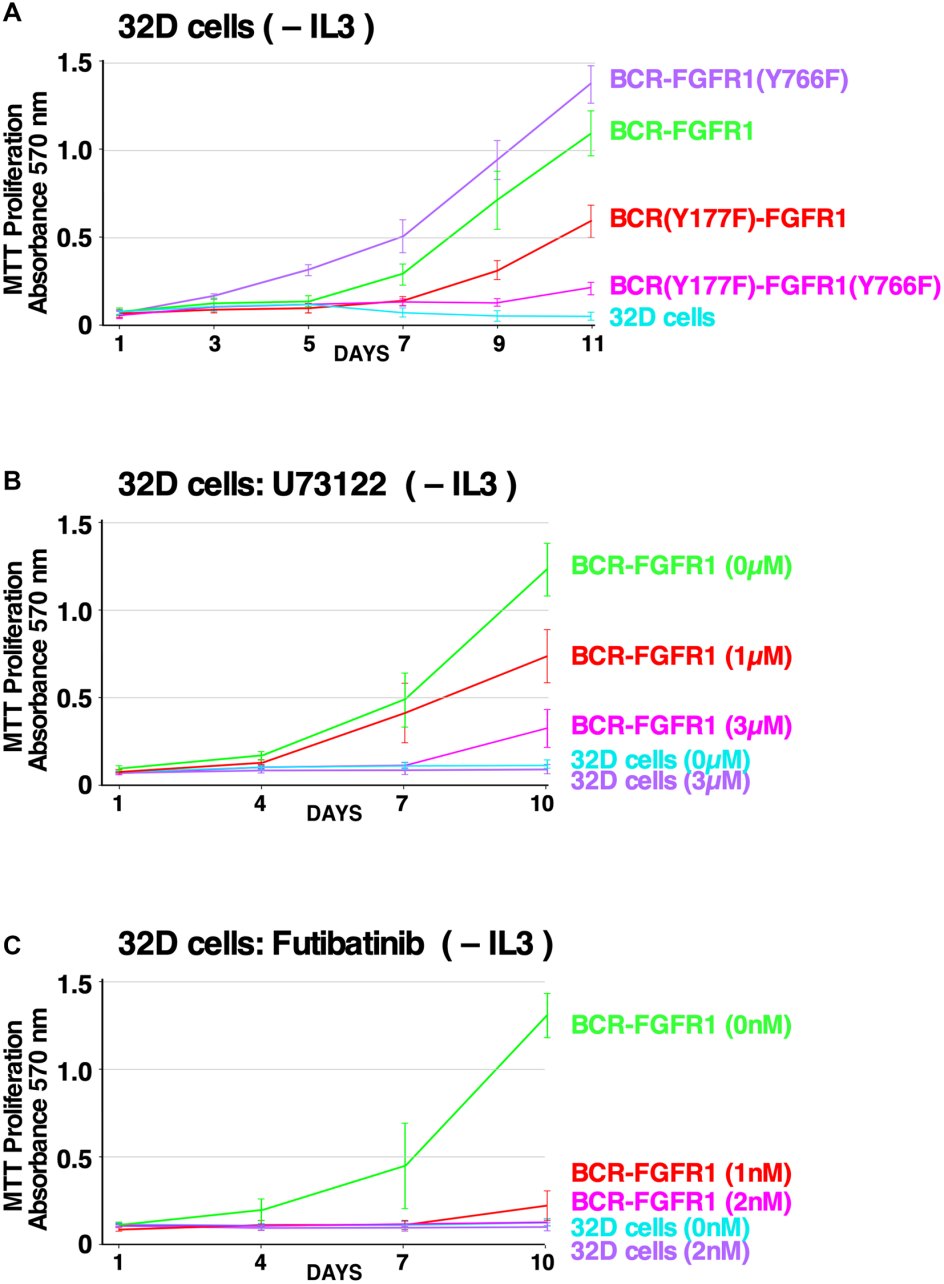
Effects of PLCγ1 and Grb2 mutations, and the efficacy of small molecule inhibitors, on hematopoietic cell proliferation. (**A**) 32D cell proliferation assay measured by MTT metabolic activity is presented using 32D cell lines stably expressing the BCR-FGFR1 fusion protein or BCR-FGFR1 derivatives, grown in the absence of IL-3 over a period of 11 days. As a control, all cell lines proliferated equally well in the presence of IL-3 over a period of 11 days (data not shown). Each experiment was performed a minimum of 3 times. (**B**) 32D cells stably expressing BCR-FGFR1 or control 32D cells were grown in the absence of IL-3, treated with increasing concentrations of the PLCγ1 inhibitor U73122 and assessed for metabolic activity by MTT assay. Each experiment was performed a minimum of 3 times, and standard error of the mean (SEM) is shown. As a control, all cell lines proliferated equally well in the presence of IL-3 (data not shown). (**C**) 32D cells stably expressing BCR-FGFR1 or control 32D cells were grown in the absence of IL-3, treated with increasing concentrations of the FGFR inhibitor futibatinib and assessed for metabolic activity by MTT assay. Each experiment was performed a minimum of 3 times, and standard error of the mean (SEM) is shown. As a control, all cell lines proliferated equally well in the presence of IL-3 (data not shown).

To confirm the results presented in Figure 1, showing the effects of PLCγ1 and Grb2 mutations on NIH3T3 cell transformation, we wished to examine the biological effects of these mutations using a more relevant hematopoietic cell line. Previous studies have utilized either Ba/F3 or 32D hematopoietic cell lines to demonstrate oncogenic and proliferative potential in these IL-3 dependent cell lines [9, 12, 32, 33]. Using 32D cells, expression of the double mutant BCR(Y177F)-FGFR1(Y766F) was unable to drive proliferation in the absence of IL-3 (Figure 5A). In contrast, cells expressing the single mutant BCR(Y766F)-FGFR1 proliferated as well or better than BCR-FGFR1-expressing cells, while cells expressing the Grb2 site single mutant, BCR(Y177F)-FGFR1, exhibited reduced but significant proliferative ability. These data demonstrate that inhibition of either signaling pathway alone fails to inhibit hematopoietic cell proliferation, and demonstrate a dual requirement for Grb2 and PLCγ1 interactions with BCR-FGFR1 for proliferation.

### PLCγ1: a potential therapeutic target for BCR-FGFR1-driven hematologic malignancies

Phospholipase C (PLC) enzymes are known for their role in cell signaling, specifically, PLCγ1 activation induces the hydrolysis of phosphatidylinositol 4,5-bisphosphate (PIP_2_), leading to the production of secondary messengers diacyl glycerol (DAG) and inositol-1,4,5-trisphosphate (IP_3_), eventually causing cell proliferation [11, 34, 35]. To probe PLCγ1 as a therapeutic target for BCR-FGFR1-driven SCLL, 32D cells stably expressing BCR-FGFR1 were treated with U73122, a small molecule PLCγ inhibitor [36], and assayed for metabolic activity. Cells expressing BCR-FGFR1 exhibited a dose-dependent response to U73122 treatment in the absence of IL-3 (Figure 5B).

### Futibatinib inhibits BCR-FGFR1 and BCR-FGFR1(K656E)-driven cell proliferation

Tyrosine kinase inhibitor (TKI) therapy is often prescribed to patients with FGFR fusions, however, while ATP-competitive FGFR inhibitors can deter tumor growth, patients commonly develop secondary kinase domain resistance mechanisms in response [37, 38]. Futibatinib (TAS-120) is a non-ATP competitive irreversible pan-FGFR inhibitor which binds to covalently to a conserved cysteine in the P-loop of the kinase domain [38]. Furthermore, futibatinib has demonstrated clinical efficacy in patients harboring FGFR2-fusion-driven cholangiocarcinoma, and is in clinical trials to assess its efficacy in the treatment of solid or myeloid and lymphoid neoplasms with FGFR1 re-arrangements (NCT04189445) [38]. 32D cells stably expressing BCR-FGFR1 were treated with increasing concentrations of futibatinib, in the absence of IL-3 (Figure 5C), and exhibited a dose-dependent response to futibatinib treatment.

## DISCUSSION

### BCR-FGFR1 exhibits absolute requirement for both Grb2 and PLCγ1

Since the discovery of BCR-ABL, over 500 additional oncogenic fusion proteins have been identified as drivers of hematologic malignancies, emphasizing the importance of characterizing these drivers and their respective cancers [9]. While FGFR2 alterations and FGFR2 fusion proteins have been identified as drivers of intrahepatic cholangiocarcinoma [13, 38, 39], FGFR1 fusion proteins are implicated as drivers of stem cell leukemia/lymphoma. The use of TKI therapy treatment often results in acquired drug resistance in patients, often through secondary kinase-activating mutations, highlighting the need to develop alternative treatments [37].

**Figure 6:**
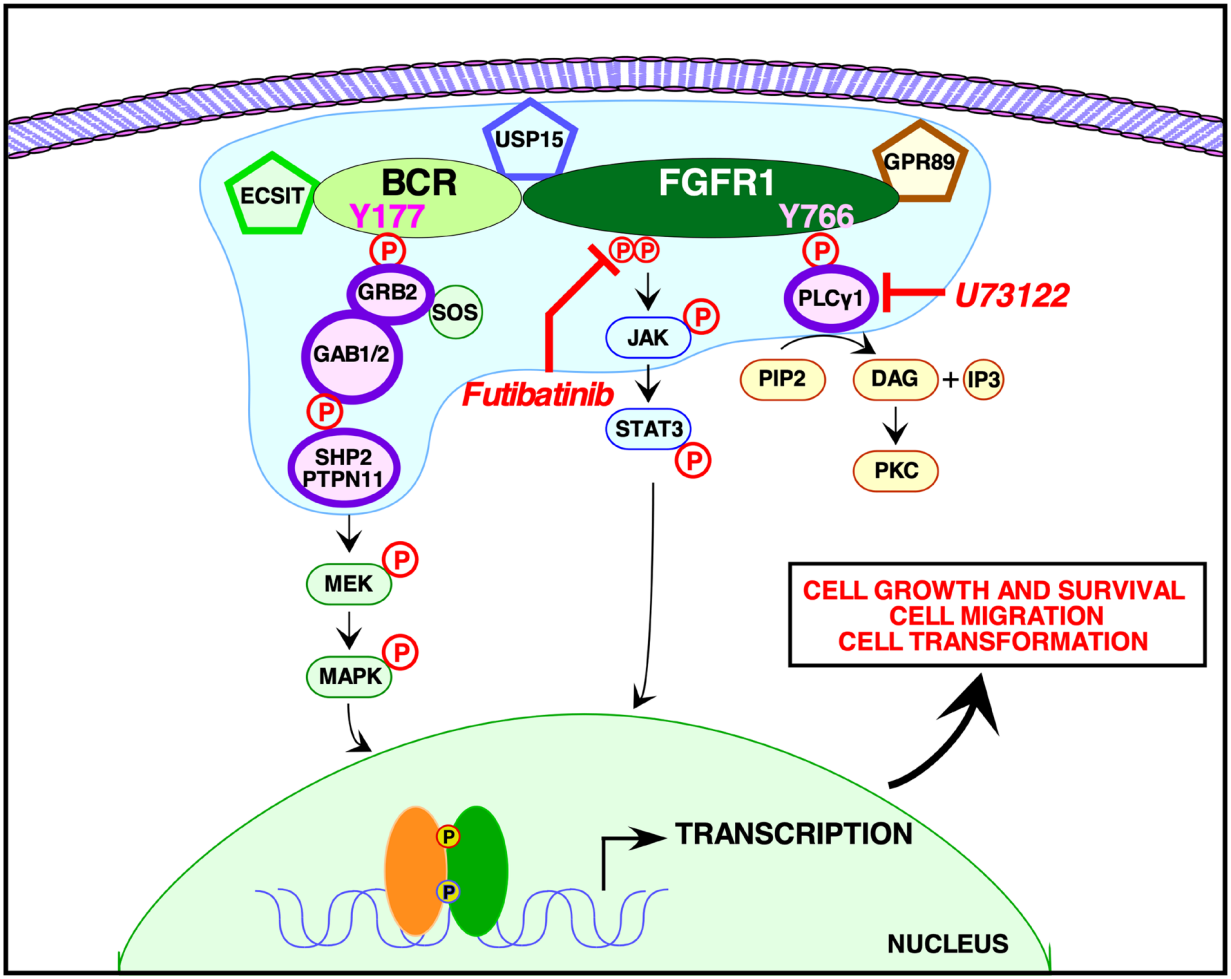
Signaling pathways activated by BCR-FGFR1. A model is presented for signaling by BCR-FGFR1 as mediated by the phosphorylated Y177 binding site for the adapter protein, Grb2, within the BCR domain of the oncogenic fusion protein, and by the phosphorylated Y766 binding site for the membrane associated enzyme, PLCγ1, within the FGFR1 domain. The proposed membrane-less protein granule [41] is represented in blue, containing the additional proteins found from our mass spectrometry interactome screen. These proteins include: Shp2 (PTPN11), Grb2, PLCγ, Usp15, Gpr89, and ECSIT. Small molecule inhibitors of PLCγ, such as U73122, used in conjunction with FGFR1 inhibitors such as the irreversible TKI futibatinib, are able to efficiently abrogate the proliferative and oncogenic effects of the BCR-FGFR1 fusion protein.

We demonstrate here that BCR-FGFR1 relies dually on the small adapter protein, Grb2, and the phospholipase, PLCγ1, for biological activity and the activation of cell signaling pathways (summarized in Figure 6). Previous work demonstrated the dependence of BCR-FGFR1 on Grb2 for CML-like leukemia, and the importance of PLCγ1 for ZNF198-FGFR1-driven EMS like disease [10]. Mutation of the Grb2 and PLCγ1 phospho-acceptor sites in BCR-FGFR1 abolished cell transformation ability and cell proliferation (Figures 1 and 5). While single mutations of either the Grb2 interaction site (Y177F in BCR) or PLCγ1 interaction site (Y766F in FGFR1) reduced biological activity, both mutations were necessary for ablation of BCR-FGFR1-driven cell proliferation. Importantly, the BCR(Y177F)-FGFR1(Y766F) double-mutant, despite being biologically inactive, retains tyrosine kinase activity; this demonstrates clearly that kinase activation alone is insufficient for biological transformation (Figure 2). Furthermore, addition of a secondary K656E kinase-activating mutation in BCR-FGFR1 did not overcome the dual requirement for Grb2 and PLCγ1 interaction for biological activity.

Our novel proteomic screen reveals for the first time the BCR-FGFR1 protein interactome, phospho-proteome, and total proteome (Figures 3 and 4). These data confirm that Grb2 and PLCγ1 interactions are necessary for BCR-FGFR1 mediated cell proliferation and identify Gab1 and PTPN11 as possible downstream effectors of Grb2 and PLCγ1 (Figure 3). Importantly, PTPN11(Shp2) inhibition has recently emerged as a therapeutic target in multiple cancer models [26, 40]. A recent study has demonstrated that certain RTK fusion proteins have the ability to assemble into higher order membraneless protein granules, which activate Ras/MAPK signaling in a ligand independent manner [41]. Interestingly, Grb2, PLCγ1, PTPN11(Shp2) and Gab1 were all enriched in these RTK protein granules, suggesting that BCR-FGFR1 may also function in the same modality, with the additional identified proteins, USP15, GPR89, and ECSIT (Figure 6).

Recently, PLCγ1 inhibition has emerged as a therapeutic target for hematologic cancers and PLCγ1 phosphorylation status is a biomarker for metastatic risk in luminal breast cancer [11, 42, 43]. However, the importance of PLCγ1 for SCLL remained uncharacterized prior to this study. While this work clearly shows the importance of PLCγ1 for BCR-FGFR1-driven SCLL, through cell-based assays and quantitative proteomics, we further demonstrate that PLCγ1 inhibition reduces overall biological activity as seen through the assays performed with U73122 (Figure 5). U73122, a known inhibitor of PLCγ1, was able to drastically decrease the biological activity of BCR-FGFR1, or of the kinase-activated BCR-FGFR1(K656E) mutant. These experiments yielded unequivocal results using NIH3T3 cell transformation assays, and of greater relevance to hematopoietic cancers, using the hematopoietic IL3-dependent cell line 32D. However, further experiments will be required examining PLCγ1 inhibitors in patient-derived cell lines and clinical studies to fully understand the efficacy of inhibiting this pathway.

ATP-competitive TKIs allow durable responses in patients with FGFR-driven tumors [44]. However, patients often develop acquired resistance to these inhibitors through the emergence of secondary kinase-activating mutations, as observed in FGFR2 fusion-driven intrahepatic cholangiocarcinoma [13, 38]. Futibatinib, a non-ATP competitive irreversible pan-FGFR inhibitor, reduces BCR-FGFR1 and BCR-FGFR1(K656E)-driven cell transformation and cell signaling in a dose-dependent manner (Figure 5). Furthermore, futibatinib treatment resulted in a durable complete hematologic and cytogenetic remission in a patient with PCM1-FGFR1 positive myeloid neoplasm. This demonstrates that futibatinib may be efficacious in treating BCR-FGFR1-driven SCLL to overcome additional kinase-activating mutations.

### Implications for additional hematological cancers

Since the detection of BCR-ABL, BCR has been identified as a commonly occurring fusion partner in many other hematologic malignancies. Notably, BCR-PDGFRA, BCR-JAK2, and BCR-RET fusions have been established as additional drivers of myeloid and lymphoid neoplasms, while BCR-NTRK2 was identified as a potential driver of glioblastoma [45, 46]. Clinical evidence suggests that patients who harbor these mutations benefit from personalized therapies, highlighting the importance of molecular testing and oncoprotein characterization. Identified BCR fusion proteins in patients contain at minimum the coiled-coil oligomerization domain and Grb2 biding site contributed by BCR, fused to a constitutively activated tyrosine kinase contributed by a partner gene [45]. Due to many structural similarities between these identified fusion oncogenes, the results described in this study may be applicable to additional leukemias driven by BCR fusion proteins.

The quantitative proteomic profiling described here detected Shp2 and Gab1 as possible downstream effectors of Grb2 in BCR-FGFR1-induced malignancies. While Shp2 is essential in driving BCR-ABL mediated leukemogenesis [47], our results suggest that Shp2 also plays a vital role in BCR-FGFR1 driven hematologic malignancies. As the Grb2 binding site at Tyr177 in BCR is uniformly conserved among other BCR-fusion proteins, such as BCR-JAK2, BCR-PDGFRA, BCR-RET and BCR-NTRK2, our results suggest that Shp2 and Gab1 play an equally important role in cancers driven by these oncogenes as well. Furthermore, inhibition of Shp2 maybe beneficial for these BCR-fusion protein driven hematologic cancers, however, this remains to be investigated.

### PLCγ1: an emerging target for myeloid and lymphoid neoplasms

The membrane associated phospho-enzyme, PLCγ1, is typically activated by RTKs and mediates downstream signaling and cell proliferation. However, PLCγ1 is overexpressed and mutated in various cancers including breast cancer, gastric cancer, colorectal cancer, T-cell lymphoma, and AML [11, 48]. Activation of this enzyme is associated with cancer cell migration and metastasis, which has resulted in PLCγ1 emerging as a potential therapeutic target for cancer treatment [11, 48]. In hematological malignancies, PLCγ1 is known to play an important role in AML leukemogenesis and is required for AML1-ETO induced leukemic stem cell survival; however, the role of PLCγ1 in SCLL was unknown prior to this study [11, 49]. Through this work, we demonstrate that PLCγ1 is required for BCR-FGFR1-induced cell proliferation and establish PLCγ1 inhibition as a potential therapeutic target for SCLL. Furthermore, PLCγ1 inhibition may emerge as an alternative therapeutic option for imatinib-resistant CML cases.

Stem cell leukemia/lymphoma (SCLL) exhibits distinct clinical and pathological features, characterized by chromosomal translocations involving the FGFR1 gene at chromosome 8p11. Currently, 15 FGFR1 partner genes have been identified in SCLL, all of which contain a crucial dimerization domain, imperative for FGFR1 tyrosine kinase activity [4, 7]. Due to the large number of FGFR1 partner genes, each with its own specific dimerization domain, inhibition of oligomerization or dimerization may not be easily feasible as a therapeutic modality for SCLL. However, all identified FGFR1 fusions in SCLL display a commonality in containing a PLCγ1 binding site at the C-terminus of FGFR1 at Tyr766 [4]. Due to this similarity across these FGFR1 fusions, PLCγ1 inhibition may be a beneficial therapeutic target in treating FGFR1 translocation induced myeloproliferative neoplasms.

The characterization of driver mutations in cancer is imperative, as this provides a mechanistic understanding of cancer progression. SCLL patients have a median one-year overall survival rate of 43%. This poor prognosis and lack of molecular targeted therapies highlights SCLL as a critically unmet medical need. This study provides new information concerning the dual roles of Grb2 and PLCγ1 as modulators in BCR-FGFR1-driven SCLL. Our use of quantitative mass spectrometry methods unraveled the BCR-FGFR1 mediated protein interactome and protein phospho-proteome. This comprehensive screen identified Shp2, Gab1, GPR89, USP15, and ECSIT as new proteins for further study, as they may be key effectors in hematopoietic transformation exploited by BCR-FGFR1. With the advent of personalized medicine, the characterization of oncogenic fusion proteins resultant from chromosomal translocations provides opportunity to introduce molecular therapies. Our work highlights the importance of sequencing based, mutation-specific therapies for FGFR1 induced hematologic malignancies.

## MATERIALS AND METHODS

### DNA constructs

The mutations for BCR(Y177F), FGFR1(766F), and all other mutations described were introduced by PCR-based site-directed mutagenesis. Other clones were as previously described [9].

### Cell culture and immunoblotting

HEK293T cells, 32D cells (clone 3) (ATCC CRL-11346) cells, and NIH3T3 cells were maintained as described previously [9, 13]. See Supplementary Materials for complete information, including HEK293T transfections, NIH3T3 transformations, and immunoblotting [50, 51].

### U73122 and futibatinib experiments

U73122 was obtained from Selleckchem (Houston, TX, USA) and futibatinib (TAS-120) was obtained from Chemgood (Glen Allen, VA, USA). Approximately 24 h after transfection, cells were starved with no FBS for 18 h. Stated concentrations of U73122 or futibatinib were added 14 h into the starvation period. Cells were then collected and lysed as described for immunoblotting and immunoprecipitation analyses. For experiments involving U73122 or futibatinib performed in NIH3T3 cell focus assays, cells were re-fed with the respective drug in 2.5% CS/DMEM media every 3–4 days, after which they were fixed and scored for transfection efficiency as described. The amount of drug was initially titrated for each assay in order to avoid toxicity to the various cell lines. Each experiment had a total of 2 technical replicates and 4 biological replicates.

### Mass spectrometry sample preparation

HEK293T cells were plated one day prior to transfection at 3.0 × 10^6^ cells per 15 cm tissue culture plate. Five plates per sample were transfected with BCR-FGFR1, BCR-FGFR1(K514A), BCR(Y177F)-FGFR1(Y766F) or BCR(Y177F)-FGFR1(K656E/Y766F). Each plate was transfected with 10 μg of each respective pcDNA3 plasmid construct. A total of four biological replicates were generated.

Following cell lysis and protein digestion, peptides were labeled with Tandem Mass Tags (TMT) and fractionated by high pH reversed phase chromatography. The subsequent TMT-labeled phosphopeptides were sequentially enriched by Immobilized Metal Affinity Chromatography (IMAC) and anti-phospho-Tyrosine antibody. All mass spectra were analyzed with Spectromine software [52, 53]. Statistical analyses of TMT total and phosphoproteome data were carried out separately using in-house R script (version 3.5.1, 64-bit), including R Bioconductor packages limma (Linear Models for Microarray Data) [53], ssGSEA [54] and MSstatsTMT (Mass Spectrometry statistical package) [27]. All gene ontology analyses functions in Tables 1B and 1C had a minimum *p*-value of 1.0 × 10^−3^ and a minimum of three protein hits per GO function. All determined phospho-sites in Table 1A had a minimum log 2-fold change in phosphorylation compared to the control samples, and a minimum *p*-value of 1.0 × 10^−5^. See Supplementary Information for detailed methods.

## SUPPLEMENTARY MATERIALS



## Abbreviations

32D: murine myeloblast-like IL3-dependent hematopoietic cell line
AML: Acute Myeloid Leukemia
BCR: Breakpoint Cluster Region
CML: Chronic Myeloid Leukemia
ECSIT: Evolutionarily Conserved Signaling Intermediate In Toll
EMS: 8p11 Myeloproliferative Syndrome
FGFR1: Fibroblast Growth Factor Receptor 1
FGFRs: Fibroblast Growth Factor Receptors
Gab1: GRB2 Associated Binding Protein 1
GO function: Gene Ontology function
GPR89: G Protein-Coupled Receptor 89B
Grb2: Growth Factor Receptor Bound protein 2
IMAC: Immobilized Metal Affinity Chromatography
LC-MS/MS: Liquid Chromatography with tandem mass spectrometry
MTT: 3-(4,5-Dimethylthiazol-2-yl)-2,5-diphenyltetrazolium bromide
PLCγ1: Phospholipase C Gamma 1
PTPN11: Protein Tyrosine Phosphatase Non-Receptor Type 11
RTK: Receptor Tyrosine Kinase
SCLL: Stem Cell Leukemia/Lymphoma
SEM: Standard Error of the Mean
Shp2: SH2 Domain-Containing Protein Tyrosine Phosphatase 2
SOS: Son of Sevenless
STAT3: Signal Transducer And Activator Of Transcription 3
T-ALL: T-cell Acute Lymphoblastic Leukemia Lymphoma
TAP: Tandem Affinity Purification
TKI: Tyrosine Kinase Inhibitor
TMT: Tandem Mass Tag
U73122: PLCγ1 inhibitor, 1-(6-((3-Methoxyestra-1,3,5(10)-trien-17-yl)amino)hexyl)-1H-pyrrole-2,5-dione
USP15: Ubiquitin Specific Peptidase 15
