# Supplementary material for: Proteomic analysis reveals dual requirement for Grb2 and PLCγ1 interactions for BCR-FGFR1-Driven 8p11 cell proliferation
Source: Oncotarget. 2022 May 11;13:659–76. doi: 10.18632/oncotarget.28228 (PMC9093983; doi:10.18632/oncotarget.28228)
Supplement: Supplementary file 1 [file oncotarget-13-28228-s001.pdf]

Proteomic analysis reveals dual requirement for Grb2 and PLCγ1 interactions for BCR-FGFR1-Driven 8p11 cell proliferation

SUPPLEMENTARY MATERIALS

Cell transfection, immunoprecipitation, immunoblot analysis

HEK293T cells were transfected with 3 µg of the pcDNA3 plasmid constructs using calcium phosphate transfection as described [1]. Approximately 24 h after transfection, cells were starved with no FBS for 18 h. Cells were washed in ice-cold PBS and lysed in radioimmunoprecipitation assay buffer [RIPA; 50 mM Tris-HCl (pH 8.0), 150 mM NaCl, 1% TritionX-100, 0.5% sodium deoxycholate, 0.1% SDS, 50 mM NaF, 1 mM sodium orthovanadate, 1 mM PMSF, and 10 µg/mL aprotinin]. Lowry assay was used to measure total protein concentration. For immunoprecipitation, either Grb2 (C-23) or FGFR1 antibody (D8E4) was added to lysates for overnight incubation at 4°C with rocking, immune complexes were bound to Protein A-Sepharose (MilliporeSigma, Burlington, MA, USA) and washed extensively with RIPA buffer. Samples were separated by 10% or 12.5% SDS-PAGE and transferred to Immobilon-P membranes (MilliporeSigma, Burlington, MA, USA). Immunoblotting was performed as described [2].

NIH3T3 cells were transfected with 10 µg of the pLXSN plasmid constructs using Lipofectamine 2000 reagent (Invitrogen, Waltham, MA, USA). Approximately 14 h after transfection, media was replaced with fresh media. Transfected cells were split into +/−G418 plates and refed with either 10% CS/DMEM +G418 or with 2.5% CS/DMEM for two times a week for 14 days following cell splitting [3]. Number of foci were, normalized by transfection efficiency, and quantitated relative to a positive control +/−SEM. All assays were performed a minimum of 3 times.

Interleukin-3 (IL-3) independent growth in 32D cells

A total of 1 × 10^6 32D cells were electroporated (1,500V, 10 ms, 3 pulses) by Neon Transfection system using 30 µg of BCR-FGFR1 or its derivatives in pLXSN in triplicate. 48 h after transfection, cells were selected with 1.5 mg/mL G418 for 10 days to generate stable cell lines before starting IL-3 independent growth assays. Triplicate flasks were seeded with the cell lines at 4 × 10^4 cells/ml in the presence or absence of mouse IL-3. On noted days, samples were counted and measured for MTT metabolic activity as described [3]. For U73122 and futibatinib treatment, cells were seeded with the stated concentrations of drug –/+ IL-3. MTT metabolic activity was measured on days 1, 4, 7, and 10. A concentration of 5 µM or higher of U73122 was toxic to 32D cells in the presence of IL-3.

Antibodies and reagents

Antibodies were obtained from the following sources: BCR (B-12), Grb2 (C-23), STAT3 (C-20), from Santa Cruz Biotechnology (Dallas, TX, USA); FGFR1 (D8E4), phospho-STAT3 (Tyr705; D3A7), phospho-p44/42 MAPK (Erk1/2) (Thr202/Tyr204) (D13.14.4E) PLC gamma (2822) and phospho-PLC gamma (2821) from Cell Signaling (Danvers, MA, USA); horseradish peroxidase (HRP) anti-mouse, HRP anti-rabbit, and Enhanced chemiluminence (ECL and Prime-ECL) reagents were from GE Healthcare (Little Chalfont, UK). MG132, and recombinant mouse IL-3 were obtained from R&D Systems (Minneapolis, MN, USA). Pierce protein A/G magnetic beads were from Thermo Scientific (Waltham, MA, USA), protein sepharose A from MilliporeSigma (Burlington, MA, USA), Geneticin (G418) was from Gibco (Waltham, MA, USA), and Lipofectamine 2000 Reagent was from Invitrogen (Carlsbad, CA, USA). U73122 was from Selleck Chemicals (Houston, TX, USA), and futibatinib (TAS-120) was from Chemgood (Glen Allen, VA, USA).

Mass spectrometry analysis: sample preparation for total and phosphoproteomics

HEK293T cells were plated one day prior to transfection at 3.0 × 10^6 cells per 15 cm tissue culture plate. 3 plates per sample were transfected with BCR-FGFR1, BCR-FGFR1(K514A), BCR(Y177F)-FGFR1(Y766F) or BCR(Y177F)-FGFR1(K656E/Y766F). Each plate was transfected with 10 µg of each respective pcDNA3 plasmid construct. A total of four biological replicates was used for each sample to ensure robust statistical analysis. Cells were deprived of FBS for 18–20 h prior to collection. Cells were treated with 10 µM MG132 for approximately 5 h prior to collection.

A total of 15.0 × 10^6 cells/sample were collected in ice-cold PBS, and were lysed in 8 M urea, 50 mM ammonium bicarbonate (ABC) and Benzonase, and the lysate was centrifuged at 14,000 g for 15 minutes to remove cellular debris. Supernatant protein concentration was determined using a bicinchoninic acid (BCA) protein

assay (Thermo Scientific). Prior to protein digestion, disulfide bridges were reduced with 5 mM tris(2-carboxyethyl)phosphine (TCEP) at 30°C for 60 min, and cysteines were subsequently alkylated with 15 mM iodoacetamide (IAA) in the dark at room temperature for 30 min. Urea was then diluted to 1 M urea using 50 mM ABC, and proteins were subjected to overnight digestion with mass spec grade Trypsin/Lys-C mix (Promega). Following digestion, samples were acidified with formic acid (FA) and subsequently peptides were desalted using AssayMap C18 cartridges mounted on an AssayMap Bravo Platform (Agilent Technologies).

Total peptide amount was determined using a NanoDrop spectrophotometer (Thermo Scientific) for Tandem Mass Tag (TMT) labeling at a 4:1 TMT-to-peptide ratio. Briefly, peptides were resuspended in 50% acetonitrile in 50 mM HEPES (pH 8.5) containing one of the TMTpro tags from the TMTpro 16plex reagent (Thermo Fisher). Peptide-TMT mixture was incubated for 1 h at 25°C and 600 rpm, and the reaction was stopped by addition of hydroxylamine to a final concentration of 0.4% and incubated for 15 min at 25°C and 600 rpm. TMT samples were pooled and dried using a SpeedVac system, then resuspended in 0.1% FA and desalted using a C18 TopTip (PolyLC, Columbia, MD, USA) according to the manufacturer's recommendation, and finally the organic solvent was removed in a SpeedVac system. Dried pooled sample was reconstituted in 20 mM ammonium formate pH ~10, and separated in 2 aliquots of 0.2 and 4 milligrams for total proteomics and phosphoproteomics fractionation, respectively.

Total proteome TMT aliquot (0.2 mg) was fractionated using a Waters Acquity BEH C18 column (2.1 × 15 cm, 1.7 µm pore size) mounted on an M-Class Ultra Performance Liquid Chromatography (UPLC) system (Waters). Peptides were then separated using a 35-min gradient: 5% to 18% B in 3 min, 18% to 36% B in 20 min, 36% to 46% B in 2 min, 46% to 60% B in 5 min, and 60% to 70% B in 5 min (A = 20 mM ammonium formate, pH 10; B = 100% acetonitrile (ACN)). A total of 48 fractions were collected and pooled in a non-contiguous manner into 24 total fractions. Pooled fractions were dried to completeness in a SpeedVac concentrator prior to mass spectrometry analysis.

Phosphoproteome TMT aliquot (4 mg) was fractionated using a Waters Acquity BEH C18 column (4.6 × 25 cm, 3.5 µm pore size) mounted on a Vanquish Horizon UPLC system (Thermo). TMT-labeled peptides were then separated using a 13-min gradient: 5% to 17% B in 0.5 min, 17% to 38% B in 8.5 min, 38% to 50% B in 3 min, and 50% to 90% B in 1 min (A = 20 mM ammonium formate, pH 10; B = 100% ACN). A total of 12 fractions were collected before drying to completeness. TMT-labeled phosphopeptides were sequentially enriched by IMAC and anti-phospho-Tyrosine antibody. First, LC-fractionated aliquots were enriched in an automated fashion using the AssayMAP Bravo Platform (Agilent Technologies). Briefly, Fe(III)-NTA cartridges (Agilent technologies) were primed with 250 µL of 0.1% trifluoroacetic acid (TFA) in acetonitrile (ACN) and equilibrated with 250 µL of IMAC loading buffer (80% ACN, 0.1% TFA). Dried samples were reconstituted in 100 µL of IMAC loading buffer and loaded onto the cartridge, which was then washed with 250 µL of IMAC loading buffer to remove background. Finally, TMT-labeled phosphopeptides were eluted with 100 µL of 1% ammonia directly into 20 µL of 10% formic acid. For the antibody-based phospho-Tyrosine pulldown, the IMAC enrichment flow-through of each fraction was collected, pooled and dried using a SpeedVac concentrator. Phospho-Tyrosine peptides were then enriched using PTMScan Phospho-Tyrosine mAb kit (Cell Signaling Technology) as per manufacturer's recommendations. Finally, all samples were dried down in a SpeedVac concentrator.

LC-MS/MS analysis for total and phosphoproteomics

For the phosphopeptide-enriched fractions, peptides were reconstituted with 2% ACN, 0.1% FA and analyzed by LC-MS/MS using a Proxeon EASY nanoLC system (Thermo Fisher Scientific) coupled to an Orbitrap Fusion Lumos mass spectrometer (Thermo Fisher Scientific). Peptides were separated using an analytical C18 Aurora column (75 µm × 250 mm, 1.6µm particles; IonOpticks) at a flow rate of 300 nL/min using a 75-min gradient: 1% to 6% B in 1 min, 6% to 23% B in 44 min, 23% to 34% B in 28 min, and 27% to 48% B in 2 min (A= FA 0.1%; B = 80% ACN: 0.1% FA). The mass spectrometer was operated in positive data-dependent acquisition mode. MS1 spectra were measured in the Orbitrap with a resolution of 60,000, at accumulation gain control (AGC) target of 4e5 with maximum injection time of 50 ms, and within a mass range from 350 to 1500 m/z. The instrument was set to run in top speed mode with 3-second cycles for the survey and the MS/MS scans. After a survey scan, tandem MS was performed on the most abundant precursors with charge state between +2 and +7 by isolating them in the quadrupole with an isolation window of 0.7 m/z. Precursors were fragmented with higher-energy collisional dissociation (HCD) with normalized collision energy of 35% and the resulting fragments were detected in the Orbitrap at 50,000 resolution, at AGC of 1e5 and maximum injection time of 86 ms. The dynamic exclusion was set to 20 sec with a 10 ppm mass tolerance around the precursor.

For the total peptide fractions, peptides were reconstituted with 2% ACN, 0.1% FA and analyzed by LC-MS/MS using a Proxeon EASY nanoLC system (Thermo Fisher Scientific) coupled to an Orbitrap Fusion Lumos mass spectrometer equipped with FAIMS Pro device (High-Field Asymmetric Waveform Ion Mobility Spectrometry, Thermo Fisher Scientific). Peptides were separated using an analytical C18 Aurora column (75 µm × 250 mm, 1.6 µm particles; IonOpticks) at a flow rate of 300 nL/min using a 100-min gradient: 1% to 6% B in 1

min, 6% to 23% B in 60 min, 23% to 34% B in 38 min, and 34% to 48% B in 1 min (A = FA 0.1%; B = 80% ACN: 0.1% FA). The mass spectrometer was operated in positive data-dependent acquisition mode, and the Thermo FAIMS Pro device was set to standard resolution with the temperature of FAIMS inner and outer electrodes set to 100°C. A three-experiment method was set up where each experiment utilized a different FAIMS Pro compensation voltage: −45, −65, and −80 Volts, and each of the three experiments had a 1 second cycle time. A high resolution MS1 scan in the Orbitrap (m/z range 350 to 1,500, 60k resolution, AGC 4e5 with maximum injection time of 50 ms, RF lens 30%) was collected in top speed mode with 1-second cycles for the survey and the MS/MS scans. For MS2 spectra, ions with charge state between +2 and +7 were isolated with the quadrupole mass filter using a 0.7 m/z isolation window, fragmented with higher-energy collisional dissociation (HCD) with normalized collision energy of 35% and the resulting fragments were detected in the Orbitrap at 50k resolution, at AGC of 5e4 and maximum injection time of 86 ms. The dynamic exclusion was set to 20 sec with a 10 ppm mass tolerance around the precursor.

Data analysis for total and phosphoproteomics

All TMT data was analyzed with SpectroMine software (Biognosys, version 2.7.210226.47784). The search criteria were set as follows: full tryptic specificity was required (cleavage after lysine or arginine residues unless followed by proline), 2 missed cleavages were allowed, TMTpro (K, N-terminal) and carbamidomethylation (C) were set as fixed modifications, while oxidation (M) and acetylation (protein N-terminal) were set as a variable modifications. In addition, for the phosphoproteome data, phosphorylation (S,T,Y) were also set as variable modifications. The false identification rate was set to 1% at PSM, peptide and protein group levels.

Statistical analysis of the TMT total and phosphoproteome data were carried out separately using in-house R script (version 3.5.1, 64-bit), including R Bioconductor packages Limma [4], ssGSEA [5], and MSstatsTMT [6]. For both datasets, TMT reporter intensities were log2-transformed and loess-normalized, using the *normalizeCyclicLoess* function from Limma package, across all samples to account for systematic errors. Following normalization, all phosphopeptide-spectrum matches (phosphoproteome data) and peptide-spectrum matches (total proteome data) with precursor interference lower than 0.7 and 0.8, respectively, were removed from the list prior to statistical test. In addition, for the total proteome data, non-protein-group-specific peptides were also removed from the final list. In the phosphoproteomic analysis, phosphosite-centric quantification and statistical testing for differential abundance were performed using MSstatsTMT bioconductor package. In the total proteome analysis, protein-centric quantification and statistical testing for differential abundance were performed also using MSstatsTMT bioconductor package to summarize peptide to protein level quantification.

Interactome analysis

HEK293T cells were plated one day prior to transfection at 3.0 × 10^6 cells per 15 cm tissue culture plate. 5 plates per sample were transfected with BCR-FGFR1, BCR-FGFR1(K514A), BCR(Y177F)-FGFR1(Y766F) or BCR(Y177F)-FGFR1(K656E/Y766F). Each plate was transfected with 10 μg of each respective pcDNA3 plasmid construct. A total of n = 4 biological replicates were used. Cells were deprived of FBS for 18–20 h prior to collection. Cells were additionally treated with 10 μM MG132 for approximately 5 h prior to collection. Cells were collected in ice-cold PBS and were lysed in tandem affinity purification buffer [TAP; 50 mM Tris, (pH 7.5), 100 mM NaCl, 5% glycerol, 0.2% NP-40, 1mM sodium orthovanadate, 1 mM PMSF, 10 μg/mL aprotinin, and 20 mM β-glycerol-phosphate]. Cell lysates were subjected to two cycles of freeze/thaw, then passed 10× through 16G needles (BD, Franklin Lakes, NJ, USA) on ice, and then 5× through 23G needles (BD, Franklin Lakes, NJ, USA) on ice. The remaining solid cell pellet was discarded, and protein concentration was determined on soluble cell lysate through Lowry assay.

15 μg of BCR anti-sera (B-12) was added to 10 mg of clarified cell lysate per sample. Samples were rocked during incubation at 4°C. Immune complexes were bound with Pierce protein A/G magnetic beads as per manufacturer's directions, and beads were then washed three times with tandem affinity purification buffer, then three times with 20 mM Tris pH7.5 + 120 mM NaCl, and frozen. After thawing, samples were washed three times with 50 mM ammonium bicarbonate buffer and proteins were then digested directly on-beads and subjected to LC-MS/MS analysis. Briefly, beads-proteins were resuspended with 8 M urea, 50 mM ammonium bicarbonate, and cysteine disulfide bonds were reduced with 10 mM tris(2-carboxyethyl)phosphine (TCEP) at 30°C for 60 min and cysteines were then alkylated with 30 mM iodoacetamide (IAA) in the dark at room temperature for 30 min. Following alkylation, urea was diluted to 1 M urea, and proteins were subjected to overnight digestion with mass spec grade Trypsin/Lys-C mix (Promega, Madison, WI, USA). Finally, beads were pulled down and the solution with peptides collected into a new tube. Digested peptides were then desalted in a Bravo AssayMap platform (Agilent) using AssayMap C18 cartridges, and dried down in a SpeedVac concentrator.

Prior to LC-MS/MS analysis, dried peptides were reconstituted with 2% ACN, 0.1% FA and concentration was determined using a NanoDrop™ spectrophometer (ThermoFisher). Samples were then analyzed by LC-MS/MS using a Proxeon EASY-nanoLC system (ThermoFisher) coupled to an Orbitrap Fusion Lumos mass spectrometer (Thermo Fisher Scientific). Peptides

were separated using an analytical C18 Aurora column (75 µm × 250 mm, 1.6 µm particles; IonOpticks) at a flow rate of 300 nL/min (60°C) using a 75-min gradient: 1% to 5% B in 1 min, 6% to 23% B in 45 min, 23% to 34% B in 28 min, and 34% to 48% B in 1 min (A = FA 0.1%; B = 80% ACN: 0.1% FA). The mass spectrometer was operated in positive data-dependent acquisition mode. MS1 spectra were measured in the Orbitrap in a mass-to-charge (*m/z*) of 375–1500 with a resolution of 60,000 at *m/z* 200. Automatic gain control target was set to 4×10^5 with a maximum injection time of 50 ms. The instrument was set to run in top speed mode with 2-second cycles for the survey and the MS/MS scans. After a survey scan, the most abundant precursors (with charge state between +2 and +7) were isolated in the quadrupole with an isolation window of 0.7 *m/z* and fragmented with HCD at 30% normalized collision energy. Fragmented precursors were detected in the ion trap as rapid scan mode with automatic gain control target set to 1×10^4 and a maximum injection time set at 35 ms. The dynamic exclusion was set to 20 seconds with a 10 ppm mass tolerance around the precursor.

All mass spectra were analyzed with MaxQuant software [4, 7] version 1.6.11.0. MS/MS spectra were searched against the *Homo sapiens* Uniprot protein sequence database (downloaded in January 2021) and GPM cRAP sequences (commonly known protein contaminants). Precursor mass tolerance was set to 20 ppm and 4.5 ppm for the first search where initial mass recalibration was completed and for the main search, respectively. Product ions were searched with a mass tolerance 0.5 Da. The maximum precursor ion charge state used for searching was 7. Carbamidomethylation of cysteine was searched as a fixed modification, while oxidation of methionine and acetylation of protein N-terminal were searched as variable modifications. Enzyme was set to trypsin in a specific mode and a maximum of two missed cleavages was allowed for searching. The target-decoy-based false discovery rate (FDR) filter for spectrum and protein identification was set to 1%.

SUPPLEMENTARY REFERENCES

1. Gallo LH, Meyer AN, Motamedchaboki K, Nelson KN, Haas M, Donoghue DJ. Novel Lys63-linked ubiquitination of IKKβ induces STAT3 signaling. Cell Cycle. 2014; 13:3964–76. https://doi.org/10.4161/15384101.2014.988026. [PubMed]
2. Meyer AN, McAndrew CW, Donoghue DJ. Nordihydroguaiaretic acid inhibits an activated fibroblast growth factor receptor 3 mutant and blocks downstream signaling in multiple myeloma cells. Cancer Res. 2008; 68:7362–70. https://doi.org/10.1158/0008-5472.CAN-08-0575. [PubMed]
3. Nelson KN, Meyer AN, Siari A, Campos AR, Motamedchaboki K, Donoghue DJ. Oncogenic Gene Fusion FGFR3-TACC3 Is Regulated by Tyrosine Phosphorylation. Mol Cancer Res. 2016; 14:458–69. https://doi.org/10.1158/1541-7786.MCR-15-0497. [PubMed]
4. Ritchie ME, Phipson B, Wu D, Hu Y, Law CW, Shi W, Smyth GK. limma powers differential expression analyses for RNA-sequencing and microarray studies. Nucleic Acids Res. 2015; 43:e47. https://doi.org/10.1093/nar/gkv007. [PubMed]
5. Krug K, Mertins P, Zhang B, Hornbeck P, Raju R, Ahmad R, Szucs M, Mundt F, Forestier D, Jane-Valbuena J, Keshishian H, Gillette MA, Tamayo P, et al. A Curated Resource for Phosphosite-specific Signature Analysis. Mol Cell Proteomics. 2019; 18:576–93. https://doi.org/10.1074/mcp.TIR118.000943. [PubMed]
6. Huang T, Choi M, Tzouros M, Golling S, Pandya NJ, Banfai B, Dunkley T, Vitek O. MSstatsTMT: Statistical Detection of Differentially Abundant Proteins in Experiments with Isobaric Labeling and Multiple Mixtures. Mol Cell Proteomics. 2020; 19:1706–23. https://doi.org/10.1074/mcp.RA120.002105. [PubMed]
7. Cox J, Mann M. MaxQuant enables high peptide identification rates, individualized p.p.b.-range mass accuracies and proteome-wide protein quantification. Nat Biotechnol. 2008; 26:1367–72. https://doi.org/10.1038/nbt.1511. [PubMed]
